# Phytochemical Composition and Biological Activities of Selected Wild Berries (*Rubus moluccanus* L.,* R. fraxinifolius* Poir., and* R. alpestris* Blume)

**DOI:** 10.1155/2016/2482930

**Published:** 2016-06-28

**Authors:** Mohd Fadzelly Abu Bakar, Nur Amalina Ismail, Azizul Isha, Angelina Lee Mei Ling

**Affiliations:** ^1^Faculty of Science, Technology and Human Development, Universiti Tun Hussein Onn Malaysia (UTHM), Batu Pahat, 86400 Parit Raja, Johor, Malaysia; ^2^Institute for Tropical Biology and Conservation, Universiti Malaysia Sabah, Jalan UMS, 88400 Kota Kinabalu, Sabah, Malaysia; ^3^Centre of Research for Sustainable Uses of Natural Resources (CoR-SUNR), Universiti Tun Hussein Onn Malaysia (UTHM), Batu Pahat, 86400 Parit Raja, Johor, Malaysia; ^4^Laboratory of Natural Products, Institute of Bioscience, Universiti Putra Malaysia (UPM), 43400 Serdang, Selangor, Malaysia

## Abstract

Berries, from the genus* Rubus*, are among the vital components in a healthy diet. In this study, 80% methanol extracts from the three wild* Rubus* species (*Rubus moluccanus* L.,* Rubus fraxinifolius* Poir., and* Rubus alpestris* Blume) were evaluated for their phytochemical contents (total phenolics, flavonoid, anthocyanin, and carotenoid content), antioxidant (DPPH, FRAP, and ABTS assays), antiacetylcholinesterase, and antibacterial activities. GC-MS was used for quantification of naturally occurring phytochemicals. The results showed that* R. alpestris* contained the highest total phenolic [24.25 ± 0.1 mg gallic acid equivalent (GAE)/g] and carotenoid content [21.86 ± 0.63 mg *β*-carotene equivalents (BC)/g], as well as the highest DPPH scavenging and FRAP activities. The highest total flavonoid [18.17 ± 0.20 mg catechin equivalents (CE)/g] and anthocyanin content [36.96 ± 0.39 mg cyanidin-3-glucoside equivalents (c-3-gE)/g] have been shown by* R. moluccanus*. For antibacterial assays,* R. moluccanus* and* R. alpestris* extracts showed mild inhibition towards* Bacillus subtilis*,* Staphylococcus aureus*,* Escherichia coli*, and* Salmonella enteritidis*. Anticholinesterase activity for all extracts was in the range of 23–26%. The GC-MS analysis revealed the presence of at least 12, 21, and 7 different organic compounds in 80% methanol extracts of* R. alpestris*,* R. moluccanus*, and* R. fraxinifolius*, respectively, which might contribute to the bioactivity.

## 1. Introduction 

Berries have been traditionally used by many cultures to treat various ailments. In traditional Chinese medicine, combination of Chinese raspberry (*Rubus chingii*) and “yang” tonic was used to treat infertility, impotence, low backache, poor eyesight, and frequent urination. According to aboriginal people in Australia, berries are considered as mild laxative if eaten in large quantities [1]. Berries are among the vital components in healthy diet. Their small, soft-fleshed fruits are usually consumed in fresh, frozen, dried, and product form [2]. They have received significant attention due to their potential benefit to human health [3].

Berries extracts have been demonstrated to exert anti-inflammatory, antioxidant, anticancer, antimicrobial, anthelminthic, and anti-Alzheimer activities [4–7]. Examples of berries or brambles in Rosaceae family are yellow Himalayan raspberry (*Rubus ellipticus*), hill raspberry (*Rubus niveus*), Korean black raspberry (*Rubus coreanus*), cloudberry (*Rubus chamaemorus*), and red raspberry (*Rubus idaeus*) [8, 9]. The bioactivities shown by these species are mainly due to the occurrence of their phytochemicals. Even though these berries fall under the same genus, the phytochemicals content and biological activities exerted by the closely related species are different [6]. Phenolics profile and concentration were affected by genetic (genus, species, and cultivar/genotype) and environmental (plant maturity, plant age, growing season, and field location) factors [9–12]. Previous research has shown that black raspberries displayed the highest amount of total polyphenols, flavonoid, and anthocyanin when compared to Korean raspberries and blackberries. It is also reported that the high antioxidant and anti-inflammatory activities shown by black raspberries were contributed by their high polyphenols and anthocyanin content [4].

In Malaysia Borneo, genus* Rubus* could be found on highland area of Sabah. Generally, this genus comprises 12 subgenera and consists of 500 species [13], including few domesticated species. Based on the record by Corner and Beaman [14], more than 8 species of* Rubus* can be found above 1200 m on Mount Kinabalu, for instance,* Rubus lineatus, Rubus benguetensis, Rubus elongates,* and* Rubus rosifolius*. Other species such as* Rubus moluccanus* L.,* Rubus fraxinifolius* Poir., and* Rubus alpestris* Blume could be found in Mount Alab, Crocker Range Park, Sabah, Borneo. The diversity of wild* Rubus* species in this area has attracted our attention to further investigate the bioactivities and their chemical components. Therefore, the current study aims to investigate the antioxidant, antiacetylcholinesterase, and antibacterial activities of the selected wild* Rubus* species fruits (*R. moluccanus* L.,* R. fraxinifolius* Poir., and* R. alpestris*) as well as their phytochemicals content.

## 2. Materials and Method 

### 2.1. Plant Materials and Sample Preparation

**Figure 1:**
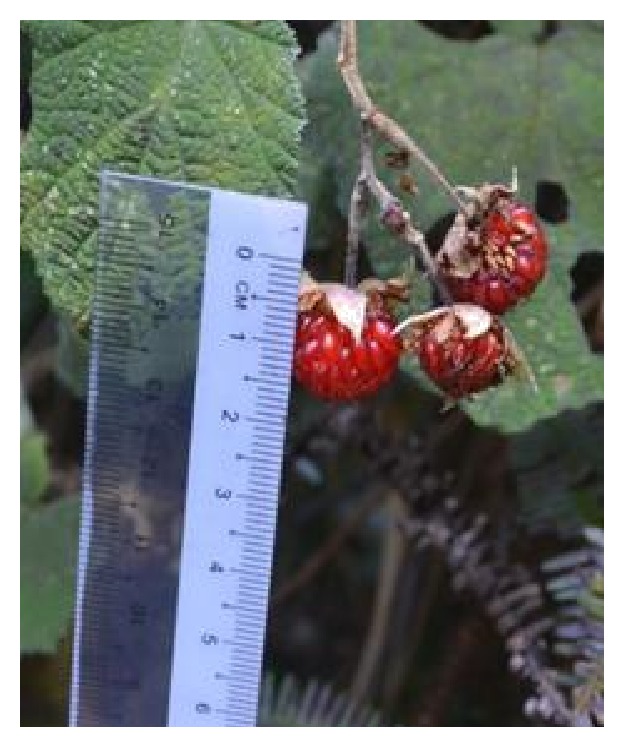
*R. moluccanus*.

**Figure 2:**
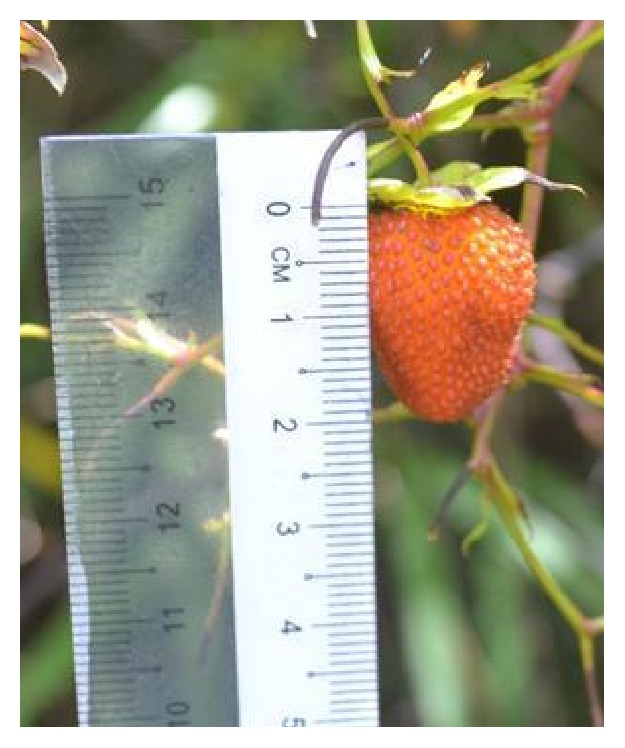
*R. fraxinifolius* .

**Figure 3:**
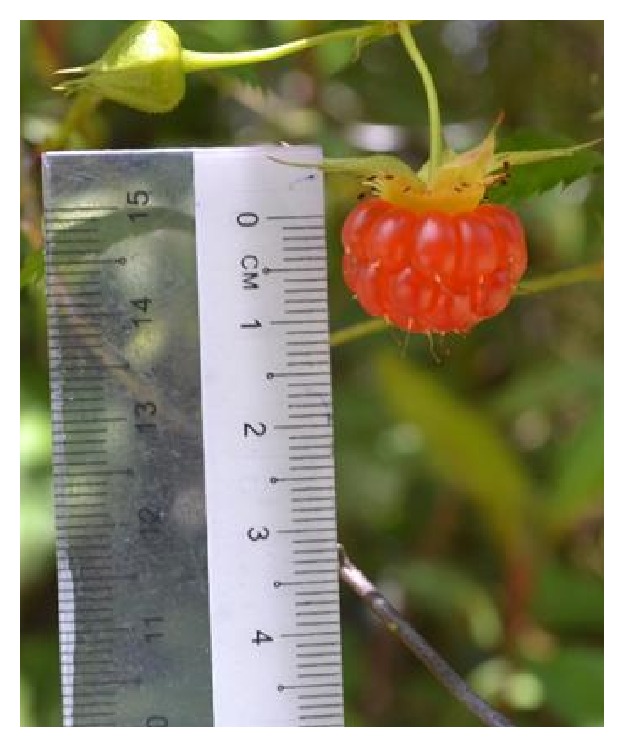
*R. alpestris*.

All samples [*R. moluccanus* (Figure 1),* R. alpestris* (Figure 2), and* R. fraxinifolius* (Figure 3)] were collected from Mount Alab, Crocker Range Park, Sabah, Malaysian Borneo, on February 2014. These plant materials were identified by a botanist, Mr. Johnny Gisil. Voucher specimens of the three samples were deposited at BORNEENSIS, Institute for Tropical Biology & Conservation, Universiti Malaysia Sabah. The fruits were cleaned, weighed, and cut into smaller pieces. Then, the fruits were kept in a freezer (−80°C) overnight before being freeze-dried for 3 days. The freeze-dried samples were ground into fine powder by using a dry grinder. The samples were sieved to get a uniform particle size and kept in an airtight container and stored in a freezer (−20°C) for further analysis.

### 2.2. Sample Extraction

Extraction method was adapted from the method described previously [15] with slight modification. About 0.1 g of freeze-dried sample was added to 30 mL of 80% (v/v) methanol. The mixture was shaken for 2 hours by using an orbital shaker set at 200 rpm at room temperature. The supernatant was decanted into a vial for further analysis.

### 2.3. Determination of Phytochemicals Content in Wild Rubus Extracts

#### 2.3.1. Total Phenolic Content

Total phenolic content was determined using Folin-Ciocalteu method [15]. About 100 *µ*L of sample extract was mixed with 0.75 mL of Folin-Ciocalteu reagent (prediluted 10 times with distilled water). The mixture was vortexed for 15 seconds. After 5 minutes, 0.75 mL of sodium bicarbonate (60 g/L) solution was added to the mixture and allowed to stand at 22°C for 90 minutes. The absorbance value was measured at 725 nm by using microplate reader. Gallic acid was used as a standard in the range of 0 to 100 *µ*g/mL and the results were expressed as mg of gallic acid equivalent in 1.0 g of dried sample (mg GAE/g). Analyses were done in triplicate for each sample.

#### 2.3.2. Total Flavonoid Content

Aluminium colorimetric method [16] was used to determine flavonoid content. Briefly, 1 mL of sample extract was added to the beaker with 4.0 mL distilled water and 0.3 mL of (5% w/v) sodium nitrite was added to the mixture. Then, 0.6 mL of (10% w/v) aluminium chloride hexahydrate was added after 5 minutes. After 6 minutes, 2.0 mL sodium hydroxide (1 M) was added to the solution and vortexed for 15 seconds. The absorbance values were measured at 510 nm by using spectrophotometer. Catechin (20–100 *µ*g/mL) was used as a standard. Results were expressed as mg catechin equivalent/g (mg CE/g) sample.

#### 2.3.3. Total Anthocyanin Content

Total anthocyanin content was measured by using a spectrophotometric pH differential protocol [17] with slight modification. Briefly, 0.5 mL extract was mixed thoroughly with 3.5 mL of potassium chloride buffer (0.025 M; pH 1.0). The mixture was mixed well with vortex and allowed to stand for 15 minutes. The absorbance values were measured at 515 and 700 nm against distilled water blank. The extract was mixed with 3.5 mL of sodium acetate buffer (0.025 M; pH 4.5) and allowed to stand for 15 minutes. The absorbance values were measured at the same wavelength. The total anthocyanins content was calculated by using the following formula:

(1) Total  anthocyanin  contentmg/g  of  dried  sample=A×Mw×DF×10ε×C,

where *A* is absorbance = (*A*_515_ − *A*_700_) pH 1.0 − (*A*_515_ − *A*_700_) pH 4.5, Mw is molecular weight for cyanidin-3-glucoside = 449.2, DF is a dilution factor of the samples, *ε* is the molar absorptivity of cyanidin-3-glucoside = 26,900, and *C* is the concentration of the buffer in mg/mL.

Results were expressed as mg cyanidin-3-glucoside equivalents (c-3-gE)/g of dried sample.

#### 2.3.4. Total Carotenoid Content

Carotenoid content in the extract was determined based on the method described previously [18]. About 150 *µ*L extract, with 150 *µ*L distilled water and 600 *µ*L methanol, was mixed in a centrifuge tube. The mixture was extracted with 300 *µ*L hexane solution and centrifuged at speed 2000 ×g for 5 minutes at 4°C. Two layers of solution were formed. The absorbance value was measured at 350 nm by using organic layer solution. *β*-Carotene was used as a reference. The result was expressed in mg BC/g sample.

#### 2.3.5. Gas Chromatography-Mass Spectroscopy (GC-MS)

Sample extracts were analysed by gas chromatography equipped with mass spectrometry (GC-MS-2010 Plus-Shimadzu). The column temperature was set to 50°C for 4 min, then increased to 320°C at the rate of 7°C/min, and then held for 20 min. The injector temperature was set at 280°C (split mode with the ratio being adjusted to 20 : 1, injection volume = 0.1 L). The flow rate of the helium carrier gas was set to 1 mL/min with a total run time of 60 min. Mass spectra were obtained from the range* m/z* 40 to 700 and the electron ionization at 70 eV. The chromatograms of the sample were identified by comparing their mass spectra with the library data (NIST 11 Library and Wiley Library) and the GC retention time against known standards.

### 2.4. Determination of Antioxidant Activities in Wild Rubus Extracts

#### 2.4.1. DPPH (2,2-Diphenyl-1-picrylhydrazyl) Radical Assay

The scavenging activity of the extract was estimated by using DPPH as a free radical model [19]. Firstly, 0.3 mM DPPH was prepared by dissolving 0.0118 g DPPH powder into 100 mL absolute methanol. Then, 1.0 mL from 0.3 mM DPPH methanol was added to 2.5 mL sample extract with the different concentration and allowed to stand for 30 minutes at room temperature in dark room. The solution was transferred into cuvette and absorbance value was measured at 518 nm by using a spectrophotometer. The blank and control absorbance value were also taken. The free radical scavenging activity was calculated by using the following formula:

(2) Scavenging  effect%=100−Abs  sample−Abs  blankAbs  control×100,

where Abs blank = 1 mL 80% (v/v) methanol + 2.5 mL extract and Abs control = 1 mL 0.3 mM DPPH methanol + 2.5 mL 80% (v/v) methanol.

The calibration curve for scavenging activity against concentration was plotted and the IC_50_ (half maximal inhibitory concentration) value was determined.

#### 2.4.2. FRAP (Ferric Reducing/Antioxidant Power) Assay

The ability of the extract to reduce ferric ion (Fe^3+^) to ferrous ion (Fe^2+^) was determined according to the previous method [20] with slight modification. FRAP reagent was prepared first. Briefly, 300 mM acetate buffer (pH 3.6) was mixed with 10 mM TPTZ and 20 mM FeCl_3_·6H_2_O with ratio 10 : 1 : 1. Then, FRAP reagent was used as a blank and was measured at 593 nm by using spectrophotometer. About 100 *µ*L sample extract and 300 *µ*L distilled water were added to the blank in test tube. After 4 minutes, second reading was taken. Fe(II) was prepared as a standard using several concentrations from 0 to 100 *µ*g/mL. A standard curve was prepared by plotting the FRAP value of each standard versus its concentration. The results were expressed as the concentration of antioxidant having a ferric reducing ability in 1 gram of sample (mM/g).

#### 2.4.3. ABTS [2,2′-Azino-bis(3-ethylbenzothiazoline-6-sulphonic acid)]

The ABTS decolorization assay was adapted from [21] with slight modification. Briefly, 7 mM of ABTS solution and 2.45 mM potassium persulfate were added to a beaker to produce blue-green color of ABTS^·+^. The mixture was allowed to stand for 16 hours in a dark room to prevent incomplete oxidation process. The mixture was diluted with 80% methanol in order to obtain absorbance of 0.7 ± 0.2 units at 734 nm. Then, 200 *µ*L extract was added to 2.0 mL ABTS^·+^ solution. The mixture was vortexed for 45 seconds and was transferred into cuvette. The absorbance value was measured at 734 nm by using a spectrophotometer. Ascorbic acid was used as a standard in the concentration range 0 to 60 *µ*g/mL. The final results were expressed as mg ascorbic acid equivalent antioxidant capacity in 1 g of sample (mg AEAC/g).

### 2.5. Determination of Acetylcholinesterase Inhibition Activity

Acetylcholinesterase (AChE) inhibition activity was determined spectrophotometrically using acetylcholine as substrate according to the method described previously [22] with slight modification. The samples with several tested concentrations were prepared separately (0–5 mg/mL). In this assay, 250 *µ*L phosphate buffer (200 mM; pH 7.7) was added to 10 *µ*L fruit extract sample. Following that, 80 *µ*L of DTNB (3.96 mg of DTNB and 1.5 mg sodium bicarbonate dissolved in 10 mL phosphate buffer pH 7.7) and 10 *µ*L enzyme (2 U/mL) were added to the mixture. The mixture was incubated for 5 minutes at room temperature (25°C). Finally, the reaction was started by adding 15 *µ*L of ATCI (the substrate that contained 10.85 mg acetylthiocholine iodide in 5 mL sodium phosphate buffer) and incubated for 5 minutes at room temperature (25°C). The color developed was measured in microtiter plate by microplate reader at 412 nm. The hydrolysis of acetylthiocholine was determined by monitoring the formation of yellow 5-thio-2-nitrobenzoate anion as a result of the reaction with DTNB with thiocholines which is catalysed by enzymes at a wavelength of 412 nm. The solvent 80% methanol was used as negative control. Donepenzil dissolved in methanol was used as standard drug at 0–5 mg/mL concentration. The percent of inhibition was calculated by using the formula below.

The percentage inhibition of acetylcholinesterase was calculated using the following formula:

(3) $$\begin{array}{c} AChE\;\;Inhibition\left(\;\%\;\right)=\left[\;\frac{\left(A_{0}-A_{1}\right)}{A_{0}}\;\right]\times 100, \end{array}$$

where *A*_0_ is the absorbance of the control (without extract) and *A*_1_ is the absorbance of the tested extract.

### 2.6. Determination of Antibacterial Activity

The antibacterial activities of the extracts were evaluated by using disc diffusion assay [23] with slight modification. A total of 0.4 mL of bacterial culture was inoculated and spread on the agar. Two Gram-positive bacteria were tested, which were* Staphylococcus aureus* and* Bacillus subtilis* and two Gram-negative bacteria,* Escherichia coli* and* Salmonella enteritidis,* were used in this study. 100 *µ*L of each sample extract was pipetted into filter paper discs (diameter: 6 mm). After drying, the filter paper discs were placed on the agar plate. Methanol was used as negative control, whereas kanamycin was used as positive control. The inhibitory activity was determined by a clear zone around the disc after incubation at 37°C for 24 h. The zone of inhibition was measured in millimeters (mm) including the disc diameter.

### 2.7. Statistical Analysis

All experiments (except for GC-MS analysis) were carried out in 3 replicates in 3 independent experiments. The result was presented as mean ± standard deviation. The data was statistically analysed by using one-way ANOVA with a significance value of *p* < 0.05 to test the significant difference between the samples. Pearson's correlation was used to determine the relationship between phytochemicals and antioxidant activity.

## 3. Results and Discussions 

### 3.1. Total Phenolic, Flavonoid, Anthocyanin, and Carotenoid Contents

Phenolics, flavonoids, anthocyanins, and carotenoids are the phytochemicals that normally presented in berries, known to possess antioxidant, anti-inflammatory, anticancer, antihypertension, antimutagenic, antineurodegenerative, and other bioactivities [4, 6, 24, 25]. Naturally, these phytochemicals are vital components for plant's physiological functions such as for pollination and protection against UV light, pathogens, and herbivore [26]. Therefore, the occurrence of the total phenolic, flavonoid, anthocyanin, and carotenoid content in the selected* Rubus* species was investigated in this study. The result (Table 1) displayed the significant differences (*p* < 0.05) among the* Rubus* species except for total carotenoid between* R. moluccanus* and* R. fraxinifolius*. The highest phenolic (24.25 ± 0.12 mg GAE/g) and carotenoid contents (21.86 ± 0.63 mg BC/g) were observed in* R. alpestris* crude extract. The highest flavonoid (18.17 ± 0.20 mg CE/g) and anthocyanin contents (36.96 ± 0.39 mg c-3-gE/g) were displayed by* R. moluccanus* fruit.

Previous studies have demonstrated the occurrence of phenolics such as ellagic acid (which normally presented as polymer of glycosylated derivative), gallic acid, chlorogenic acid, and caffeic acid in* Rubus* species [3, 8]. Both total phenolic and flavonoid contents obtained in the current study were relatively higher than other* Rubus* species in previous studies [8, 27], but slightly lower than* R. ulmifolius* methanolic extract [7]. The dark red color of fruits indicates that it might contain high level of anthocyanin and flavonoid [28] as can be seen in* R. moluccanus*. Netzel et al. [29] stated that cyanidin-3-glucoside, cyanidin-3-rutinoside, and pelargonidin-3-rutinoside are among the anthocyanins that could be found in* R. moluccanus.* The range of total anthocyanins (23.82 to 36.96 mg c-3-gE/g) of selected* Rubus* species is similar to the previously published data by Krauze-Baranowska et al. [30] on hydroethanolic extract of raspberries with the total anthocyanins between 13.0 and 88.0 mg/g dry weight. Jung et al. [4] demonstrated that* Rubus fruticosus*,* Rubus coreanus*, and* Rubus occidentalis* exerted antioxidant and anti-inflammatory activities in hydrogen peroxide and lipopolysaccharide treated RAW264.7 cells which are probably attributed to the anthocyanin content in* Rubus* species. The anthocyanins could be found on the external layer of fruit's skin cells (hypodermis), whereas the small amount of granular-form anthocyanin is deposited in the vacuole [24].

The total carotenoid content of* R. alpestris* was much higher than the quantity of carotenoid in* Rosa canina* and* Rosa rugosa* (Rosaceae family) as reported by Razungles et al. [31]. *β*-Carotene might be the major carotenoid compound in* Rubus* species as displayed by* R. chamaemorus* [32]. The variation in the phytochemicals content is due to the genetic and environmental factors [9, 10]. The species that contained most abundant polyphenols may not necessarily possess great bioactivities. This is due to the fact that polyphenols might have lower intrinsic activity and might be poorly absorbed from intestine, highly metabolized, or rapidly eliminated [27].

### 3.2. Secondary Metabolite Profiling Using GC-MS

**Figure 4:**
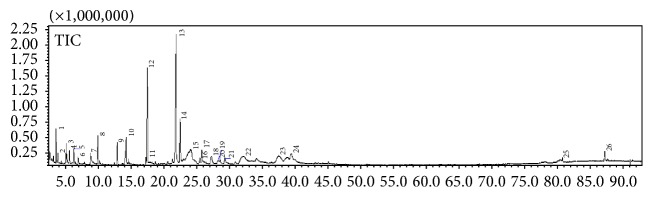
A typical gas chromatogram of the chemical constituents of* Rubus moluccanus* crude extract.

**Figure 5 fig5:**
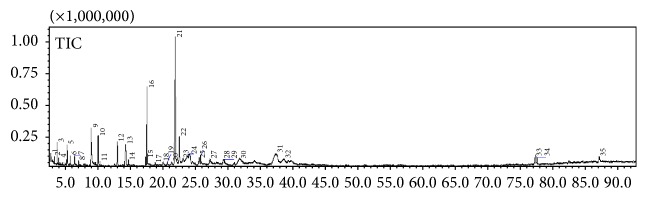
A typical gas chromatogram of the chemical constituents of* Rubus fraxinifolius* crude extract.

**Figure 6:**
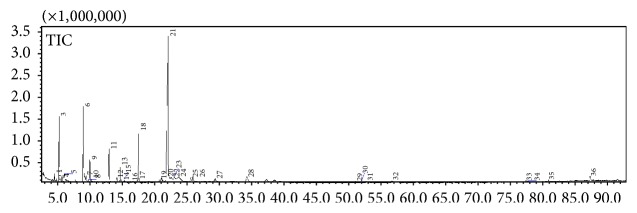
A typical gas chromatogram of the chemical constituents of* Rubus alpestris* crude extract.

GC-MS profiling was performed to identify the bioactive compounds presented in* Rubus* species. The gas chromatogram of 80%* Rubus* methanolic extracts is shown in Figures 4 [Fig fig5]–6. The analysis separated and identified a total of 21 known compounds for* R. moluccanus*, 7 known compounds for* R. fraxinifolius*, and 12 compounds for* R. alpestris* (Table 2). The major compounds in* Rubus moluccanus* included hydroxymethylfurfural (21.642%), 1,1,2-triacetoxyethane (17.908%), 2,4-dihydroxy-2,5-dimethyl-3(2H)-furan-3-one (10.345%), and 2-propenoic acid, 2-propenyl ester (6.002%). For* R. fraxinifolius*, the major compounds are 2(1H)-pyridinone, 6-hydroxy- (14.589%), 1,1,2-triacetoxyethane (10.370%), 2,4-dihydroxy-2,5-dimethyl-3(2H)-furan-3-one (8.283%), and 2-propenoic acid, 2-propenyl ester (3.589%). The major compounds in* R. alpestris* are 5-hydroxymethylfurfural (38.142%), 2(1H)-pyridinone, 6-hydroxy- (25.430%), furfural (6.6372%), and 2,4-dihydroxy-2,5-dimethyl-3(2H)-furan-3-one (5.438%).

The similar compounds that could be found in the three* Rubus* species are furfural and 2,4-dihydroxy-2,5-dimethyl-3(2H)-furan-3-one. Furfural is a precursor of furan which could be formed naturally in fruits or in processed food during thermal storage. It also acts as indicator for the occurrence of Maillard reaction [33, 34]. Furfural also is highly concentrated in berry cactus [2]. Hydroxymethylfurfural or 5-hydroxymethylfurfural (HMF) is a major compound that could be found in* R. moluccanus* and* R. alpestris*. HMF might be presented naturally in the fruit or produced at high temperature processes such as drying or during GC-MS analysis [34]. Previous study showed that 5-HMF is an antioxidative agent from* Alpinia oxyphylla* which could serve as novel therapeutic agent for Alzheimer's disease treatment and prevention [35]. In addition, HMF also has been reported to possess anticancer properties [34].

### 3.3. DPPH, FRAP, and ABTS Assays

**Figure 7:**
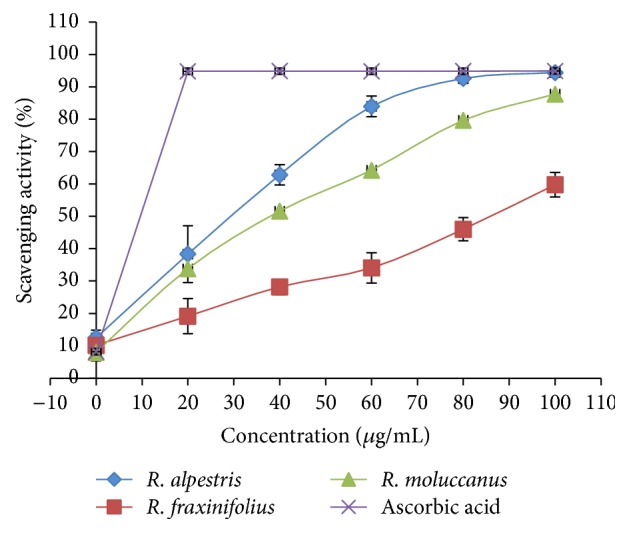
Graph of DPPH scavenging activity against concentration.

The antioxidant capacities of* Rubus* species were investigated using three different* in vitro* antioxidant assays. In DPPH assay, purple color of DPPH solution turns into yellow color in the presence of antioxidant compound [19]. The antioxidant effect of extracts on DPPH free radical was due to its hydrogen-donating ability. The DPPH scavenging activity of selected* Rubus* species and standard (ascorbic acid) was depicted in Figure 7.

The highest percentage of scavenging activity (94.36 ± 1.33%) was observed in* R. alpestris* at 100 *μ*g/mL concentration, followed by* R. moluccanus* (87.72 ± 0.71%) and* R. fraxinifolius* (59.78 ± 3.79%). However, the activities of all extracts are lesser than the ascorbic acid. The trend for IC_50_ values (Table 3) of DPPH radical scavenging activity is as follows: ascorbic acid (10.00 ± 0.58 *μ*g/mL) >* R. alpestris* (29.00 ± 3.07 *μ*g/mL) >* R. moluccanus* (38.00 ± 1.63 *μ*g/mL) >* R. fraxinifolius* (86.00 ± 3.65 *μ*g/mL). The DPPH radical scavenging activity of our investigated* Rubus* species is similar to or even better than the previous study [36]. As reported by Ahmad et al. [36], at 100 *µ*g/mL concentration,* Rubus ulmifolius* was able to inhibit 87.62%, whereas* R. ellipticus* and* R. niveus* were able to inhibit 54.82% and 74.54% DPPH free radical. This indicates that the investigated* Rubus* species in current analyses are more effective to inhibit DPPH free radical compared to other* Rubus* species.

FRAP method is the assay to determine the antioxidant capacity which involves Single Electron Transfer (SET) mechanism. In this method, ferric ion is reduced to ferrous ion at low pH, which caused colored ferrous tripyridyltriazine complex to be formed [20]. The reducing ability of* R. alpestris* (70.93 ± 6.26 mM Fe^2+^/g) is the highest compared to* R. moluccanus* (50.37 ± 5.28 mM Fe^2+^/g) and* R. fraxinifolius* (26.34 ± 4.79 mM Fe^2+^/g). Our results showed higher FRAP value as compared to* Rubus ellipticus* (3.43 mM ascorbic acid equivalent (AAE)/100 g) and* Rubus niveus* (2.06 AAE/100 g) in previous research [8].

Principally, ABTS decolorization assay is quite similar to DPPH assay, which involves the scavenging activity of the free radicals. However, the ABTS salt must be generated by enzymatic or chemical reaction first [21]. The* Rubus* fruit extracts exerted lower scavenging effects against ABTS radicals. There are no significant differences (*p* < 0.05) between the samples for ABTS assay. In comparison to the previous literature, ABTS assay in current study displayed lower value compared to* R. idaeus* cultivar which are “Autumn Bliss” and “Polka” in the range 1.94 to 2.12 mg AEAC/g [26]. In summary,* R. alpestris* revealed the highest content for phenolics and carotenoid which is in agreement with its highest antioxidant activity.

The phytocompounds (phenolics, flavonoid, anthocyanin, and carotenoid) might contribute to the antioxidant activity of the extract. Hence, correlation analyses were performed to investigate the relationship between the phytochemical compounds and antioxidant activity. Strong positive correlation has been displayed by total phenolics and DPPH scavenging activity (*r* = 0.972). The total carotenoid and anthocyanin content showed moderate positive correlation with DPPH scavenging assay (*r* = 0.761 and *r* = 0.764). However, no correlation existed between total flavonoid and DPPH scavenging activity which is in contrast with past research [7, 8, 11]. These results corroborate the previous report that proved the presence of positive correlation between phenolic and antioxidant capacity in the extracts [3, 7].

FRAP assays showed significant correlation with total phenolic, carotenoid, and anthocyanin (*p* < 0.05) with value *r* = 0.949, 0.770, and 0.745. Findings by Pantelidis et al. [27] and Deighton et al. [37] are supporting this study. Analysis on various berries species indicated that there was a strong correlation between phenol content with the FRAP assay [27, 37]. However, total anthocyanins have less linear correlation with total antioxidant capacity (*r* = 0.635) in previous research. The correlation between phytochemicals investigated in this study with ABTS assay was not significant (*p* > 0.05). Current result is in contrast with the previous study that displayed strong positive correlation between the total phenolic, flavonoid, and anthocyanin with ABTS assay [7, 8, 26].

### 3.4. Acetylcholinesterase Inhibition Activity

Several drugs for memory loss and cognitive deficits improvement are available in the market. However, these drugs possess some side effects. Phytochemicals from plants might be an alternative to be developed as a source of acetylcholinesterase inhibitors [38]. The acetylcholinesterase enzyme activity is measured based on the reduction of yellow color produced from thiocholine when it reacts with dithiobisnitrobenzoate ion [22]. The results were expressed in percentage. At the highest concentration (5 mg/mL), the fruit extracts displayed weak anticholinesterase activity. The highest anticholinesterase activity was shown by* R. moluccanus* (26.42 ± 1.41%), followed by* R. alpestris* (25.30 ± 1.56%) and* R. fraxinifolius* (23.06 ± 1.12%). Donepenzil (positive control) showed complete acetylcholinesterase inhibition activity (100%) when tested at 1 mg/mL. The inhibition of acetylcholinesterase noted in this study is very similar to the* Sanguisorba minor* aerial part and* Rosa Damascene* floret (Rosaceae family), which showed a weak inhibitory effect on acetylcholinesterase enzyme [39]. Kim et al. [38] demonstrated that* Rubus coreanus* ethanolic extract (1 mg/mL concentration) exhibits 36.60 ± 1.25% inhibitory effects on acetylcholinesterase enzyme. Active compound that was isolated from* R. coreanus* was identified as 3,4,5-trihydroxybenzoic acid (gallic acid). Thus, these phenolic acids might also contribute to the acetylcholinesterase activity of the investigated* Rubus* species in current study.

### 3.5. Antibacterial Activity

Since* Rubus* species displayed numerous secondary metabolites, the effects of the extract against some common pathogenic bacteria were investigated in this study. The result of our current research demonstrated that* R. moluccanus* and* R. alpestris* were effective against Gram-positive and Gram-negative bacteria (Table 4).* R. alpestris* showed the highest activity against* S. enteritidis* (8.50 ± 1.80 mm) followed by* B. subtilis* (7.83 ± 1.26 mm). Rauha et al. [40] have reported that* R. chamaemorus* and* R. idaeus* displayed only slight antibacterial effects against* S. aureus* and* E. coli*, whereas the inhibition towards* B. subtilis* was found to be moderate. In our study,* R. fraxinifolius* showed no inhibition towards* B. subtilis.* The variation in the inhibition activity of* Rubus* species is due to the differences in cell surface structure between the Gram-negative and Gram-positive bacteria [41].

Phenolic compounds such as flavone, quercetin, and naringenin were the potential compounds that contribute to the antibacterial activities against* S. aureus*,* B. subtilis*, and* E. coli* [40]. In addition, anthocyanidin, pelargonidin, delphinidin, cyanidin, and cyanidin-3-glucoside in berry extracts were able to inhibit the growth of* E. coli* [41]. Our findings suggested that all of the investigated* Rubus* species contained high amount of anthocyanin which might contribute to the inhibition towards* E. coli*. Puupponen-Pimiä et al. [41] demonstrated that Finnish berry extracts were more effective to inhibit the growth of Gram-negative bacteria as compared to Gram-positive bacteria. Generally,* R. alpestris* is more beneficial in treating the Gram-positive and Gram-negative bacteria. It is worth noting that the present study is only a preliminary attempt to assess the antibacterial potential of the* Rubus* species. Hence, further detailed bioassay needs to be applied for assessing the antibacterial activity.

## 4. Conclusions

Our results indicate there is a high variability in the phytochemicals content of* Rubus* species investigated which might be due to the genetic and environmental factors. Methanol-water extracts of* R. moluccanus, R. fraxinifolius*, and* R. alpestris* fruits showed a significant amount of phytochemicals, which contribute to antioxidant, antibacterial, and antiacetylcholinesterase activities.* R. alpestris* displayed the highest potential as a natural source of tested activities. Utilization of* Rubus* fruits in diet could offer health benefit to our body. Since there is less previous research on these particular species, this study might contribute to the additional data on phytochemistry and bioactivities of the genus* Rubus*.

## Figures and Tables

**Table 1:** Phytochemicals content of selected wild *Rubus* species.

Sample	Total phenolic^1^	Total flavonoid^2^	Total anthocyanin^3^	Total carotenoid^4^
*R. moluccanus*	20.76 ± 0.24^b^	18.17 ± 0.20^a^	36.96 ± 0.39^a^	9.69 ± 0.58^b^
*R. fraxinifolius*	11.09 ± 0.10^c^	5.82 ± 0.02^c^	23.82 ± 0.77^c^	10.49 ± 1.01^b^
*R. alpestris*	24.25 ± 0.12^a^	8.88 ± 0.53^b^	33.62 ± 1.39^b^	21.86 ± 0.63^a^

Values are presented as mean ± standard deviation (SD) (*n* = 3) which, with different letters (within column), are significantly different at *p* < 0.05.

^1^Total phenolic content was expressed as mg gallic acid equivalents in 1 g of dried sample (mg GAE/g).

^2^Total flavonoid content was expressed as mg catechin equivalents in 1 g of dried sample (mg CE/g).

^3^Total anthocyanin content was expressed as mg of cyanidin-3-glucoside equivalents in 1 g of dried sample (mg c-3-gE/g).

^4^Total carotenoid content was expressed as mg of *β*-carotene in 1 g of dried sample (mg BC/g dried sample).

**Table 2:** Chemical composition of different *Rubus* species, that is, *R. moluccanus, R. fraxinifolius*, and *R. alpestris*.

Sample	Number	Name of the compound	Concentration (%)	Retention time
*R. moluccanus*	1	2-Propenoic acid, 2-propenyl ester	6.002	3.693
	2	Pyruvate	2.998	3.969
	3	Furfural	3.185	5.272
	4	1,3-Butadiene-1-carboxylic acid	0.913	5.714
	5	Propenoic acid, 2-methyl-, methyl ester	2.857	6.403
	6	dl-Glyceraldehyde dimer	0.934	7.041
	7	2(1H)-Pyridinone, 6-hydroxy-	5.716	8.949
	8	2,4-Dihydroxy-2,5-dimethyl-3(2H)-furan-3-one	2.127	10.049
	9	Pentanoic acid, 4-oxo-	2.891	13.002
	10	2-Hydroxy-3-methyl-4- pyrone	7.549	14.370
	11	Isopropylmethylnitrosamine	0.922	17.391
	12	2,4-Dihydroxy-2,5-dimethyl-3(2H)-furan-3-one	10.345	17.604
	13	Hydroxy methyl furfural	21.642	21.970
	14	1,1,2-Triacetoxyethane	17.908	22.569
	18	Butanedioic acid, 2-hydroxy-2-methyl, (S)-	1.493	27.358
	20	Benzeneacetic acid, 4-hydroxy-, methyl ester	1.504	28.634
	21	Succinic acid, 3-methylbutyl pentyl ester	1.193	29.396
	23	*β*-D-Glucopyranoside, methyl	3.800	37.574
	24	Quinic acid	1.981	39.498
	25	*β*-Tocopherol	0.322	80.794
	26	*γ*-Sitosterol	0.204	87.214

*R. fraxinifolius*	3	2-Propenoic acid, 2-propenyl ester	3.589	3.685
	5	Furfural	2.503	5.261
	6	1,3-Butadiene-1-carboxylic acid	0.505	5.703
	9	2(1H)-Pyridinone, 6-hydroxy-	14.589	8.940
	16	2,4-Dihydroxy-2,5-dimethyl-3(2H)-furan-3-one	8.283	17.560
	22	1,1,2-Triacetoxyethane	10.370	22.514
	32	3-Deoxy-d-mannoic lactone	0.508	38.612

*R. alpestris*	3	Furfural	6.637	5.259
	6	2(1H)-Pyridinone, 6-hydroxy-	25.430	8.990
	9	2,4-Dihydroxy-2,5-dimethyl-3(2H)-furan-3-one	1.517	10.035
	11	Furaneol	3.325	12.979
	13	1H-Imidazole-4-carboxylic acid, methyl Ester	3.287	14.681
	18	2,4-Dihydroxy-2,5-dimethyl-3(2H)-furan-3-one	5.438	17.572
	21	5-Hydroxymethylfurfural	38.142	22.107
	27	Butane, 1,1′-1-[(Isopentyloxy)methoxy]-3-methylbutane	0.968	29.364
	28	Rhamnose	2.277	34.260
	31	5,5′-Oxy-dimethylene-bis(2-furaldehyde)	0.249	52.683
	35	*β*-Tocopherol	0.140	80.799
	36	Stigmast-5-en-3-ol	0.117	87.228

**Table 3:** Antioxidant activities of selected *Rubus* species.

Samples	DPPH assay^1^	FRAP assay^2^	ABTS assay^3^
*R. moluccanus*	38.00 ± 1.63^c^	50.37 ± 5.28^b^	0.73 ± 0.03^a^
*R. fraxinifolius*	86.00 ± 3.65^d^	26.34 ± 4.79^c^	0.75 ± 0.03^a^
*R. alpestris*	29.00 ± 3.07^b^	70.93 ± 6.26^a^	0.79 ± 0.05^a^
Ascorbic acid	10.00 ± 0.58^a^	—	—

Values are presented as mean ± SD (*n* = 3) which, with different letters (within column), are significantly different at *p* < 0.05.

^1^DPPH free radical scavenging activity represented by IC_50_ was expressed as *μ*g/mL.

^2^FRAP was expressed as mM ferric reduction to ferrous in 1 g of dry sample.

^3^ABTS free radical scavenging activity was expressed as mg ascorbic acid equivalent antioxidant capacity (AEAC) in 1 g of dry sample.

**Table 4:** Antibacterial activities of *Rubus* species against common pathogenic bacteria.

Species	Gram positive		Gram negative	
	*B. subtilis*	*S. aureus*	*E. coli*	*S. enteritidis*
*R. moluccanus*	7.33 ± 0.29^b^	7.67 ± 0.58^b^	7.00 ± 0.50^b^	7.17 ± 0.58^b^
*R. fraxinifolius*	0.00^c^	6.67 ± 1.15^b^	7.00 ± 0.87^b^	7.17 ± 1.15^b^
*R. alpestris*	7.83 ± 1.26^b^	7.33 ± 0.76^b^	7.67 ± 0.58^b^	8.50 ± 1.80^b^
Kanamycin	12.67 ± 1.53^a^	11.83 ± 0.76^a^	12.67 ± 0.58^a^	11.83 ± 0.76^a^

Values are presented as mean ± SD (*n* = 3) which, with different letters (within column), are significantly different at *p* < 0.05.
